# *In vitro* effect of direct current electrical stimulation on rat mesenchymal stem cells

**DOI:** 10.7717/peerj.2821

**Published:** 2017-01-12

**Authors:** Sahba Mobini, Liudmila Leppik, Vishnu Thottakkattumana Parameswaran, John Howard Barker

**Affiliations:** 1 Frankfurt Initiative for Regenerative Medicine, Experimental Orthopedics and Trauma Surgery, Johann Wolfgang Goethe Universität Frankfurt am Main, Frankfurt am Main, Germany; 2 School of Materials, Faculty of Engineering and Physical Sciences, University of Manchester, Manchester, United Kingdom

**Keywords:** Direct current electrical stimulation, Bone marrow-derived mesenchymal stem cells, Adipose tissue-derived mesenchymal stem cells, Bone tissue engineering

## Abstract

**Background:**

Electrical stimulation (ES) has been successfully used to treat bone defects clinically. Recently, both cellular and molecular approaches have demonstrated that ES can change cell behavior such as migration, proliferation and differentiation.

**Methods:**

In the present study we exposed rat bone marrow- (BM-) and adipose tissue- (AT-) derived mesenchymal stem cells (MSCs) to direct current electrical stimulation (DC ES) and assessed temporal changes in osteogenic differentiation. We applied 100 mV/mm of DC ES for 1 h per day for three, seven and 14 days to cells cultivated in osteogenic differentiation medium and assessed viability and calcium deposition at the different time points. In addition, expression of osteogenic genes, Runx2, Osteopontin, and Col1A2 was assessed in BM- and AT-derived MSCs at the different time points.

**Results:**

Results showed that ES changed osteogenic gene expression patterns in both BM- and AT-MSCs, and these changes differed between the two groups. In BM-MSCs, ES caused a significant increase in mRNA levels of Runx2, Osteopontin and Col1A2 at day 7, while in AT-MSCs, the increase in Runx2 and Osteopontin expression were observed after 14 days of ES.

**Discussion:**

This study shows that rat bone marrow- and adipose tissue-derived stem cells react differently to electrical stimuli, an observation that could be important for application of electrical stimulation in tissue engineering.

## Introduction

Large segment bone defects, caused by open fractures, non-unions, infections and tumor resection are a major challenge in trauma and orthopedic surgery. Complications associated with current treatments and the projected increase in the number of cases due to ageing populations in developed countries give urgency to the search for alternative treatments (Amini, Laurencin & Nukavarapu, 2012). Tissue engineering (TE) approaches that deliver osteoprogenitor cells with osteoconductive scaffolds directly into these large defects hold great potential for achieving optimal bone healing while eliminating the associated drawbacks of conventional treatments (Petite et al., 2000).

Mesenchymal stem cells (MSCs) have been shown to be an attractive cell source for clinical bone tissue engineering applications. MSCs possess a great capacity for self-renewal and multi-lineage differentiation, including osteogenic differentiation. Several *in vitro*, *in vivo* preclinical and clinical studies have shown that MSCs are able to facilitate bone mineralization (Colnot, 2011; Gamie et al., 2014). While the main source for harvesting MSCs has been primarily bone marrow (BM), they have also been isolated from other tissues such as adipose tissue (AT) (Grottkau & Lin, 2013). MSCs harvested from different sources have been reported to exhibit different proliferation and differentiation characteristics (Musina et al., 2006). Adipose tissue derived MSCs (AT-MSCs) are able to proliferate rapidly in culture, and facilitate expansion into large numbers of cells required for clinical effectiveness (Cowan et al., 2004). Recent studies confirm that AT-MSCs do differentiate into osteogenic lineage *in vitro* (Bunnell et al., 2008; De Girolamo et al., 2007; Pelto et al., 2013). However, a comparative study between AT-MSCs and BM-MSCs revealed that AT-MSCs have more capacity to proliferate and slightly less capacity for osteogenic differentiation. In addition, AT-MSCs exhibit high tolerance to serum deprivation-induced apoptosis in comparison with BM-MSCs (Peng et al., 2008). Although AT-MSCs are able to form osteoid matrix and regenerate bone *in vivo*, their capacity to differentiate into hematopoietic marrow is still in question (Robey, 2011).

Electrical stimulation (ES) has been shown to be effective in nerve and cardiac tissue engineering applications, primarily due to the electric nature of these tissues (Ghasemi-Mobarakeh et al., 2011; Tandon et al., 2009a). The long recognized piezoelectric characteristics of bone (electricity resulting from mechanical pressure), together with the known links between ES and bone growth, make bone an attractive target tissue for investigating the role of ES in bone regeneration and healing (Tofail, Zhang & Gandhi, 2011). Electrical stimulation has been successfully used to treat bone defects clinically for more than 40 years (Ryaby, 1998). Several common modes of electrical stimulation, such as pulsed electromagnetic fields, capacitive coupling and direct current (DC), have been used both experimentally and clinically to promote bone healing in different orthopedic applications (Griffin & Bayat, 2011). While the mechanism by which ES promotes bone healing is still poorly understood, recent *in vitro* studies show that bioelectrical signals play a key role in cellular pathways involved in healing (Levin, 2009). Recently, *in vitro* studies showed that DC ES, acting partially via electrochemical reaction at the cathode, alters several MSC behaviors, such as migration, proliferation, and differentiation (Hardy et al., 2015; Tandon et al., 2009b; Tsai et al., 2009; Zhao et al., 2011). Specifically, correlations between ES and the rate of osteogenic differentiation have been reported (Fukada & Yasuda, 1957; Shamos, Lavine & Shamos, 1963). Balint et al. (2013), exposed BM-MSCs to pulsed DC ES and reported significant alterations in the expression of osteogenic marker genes. Hammerick et al. (2010), exposed AT-MSCs to pulsed DC ES and observed an increase of osteoblast-specific markers, including Runx2, Osteopontin and Type I Collagen.

Recently, various different types of physical stimuli such as static magnetic fields, cyclic strain, low frequency vibration, and electric signals have been used to improve both the proliferative and the differentiation potential of stem cells. Specifically, ES has been shown to influence cell proliferation and differentiation in tissue engineering applications (Balint, Cassidy & Cartmell, 2013; Marycz et al., 2016). In our laboratory we study the possibility of combining ES with tissue-engineering methods to see if the combination can provide benefits not previously seen in either approach individually. We investigated the changes in expression of a few osteogenic differentiation key markers; Runx2, Osteopontin and Col1A2. Runt-related transcription factor-2, which is known as Runx2 (Cbfa1/PEBP2αA/AML-3/Osf2), is often referred to as the master switch of osteogenic differentiation. It interacts with the promoter regions of the key osteoblast specific genes such as osteocalcin, osteopontin, collagen I, bone sialoprotein, alkaline phosphatase and TGFβ receptor 1 (Nakamura et al., 2009). Secreted phosphoprotein 1 which is known as Osteopontin (OPN) has important role as a regulator of cytoskeleton dynamics and gene expression. In bone remodeling, up-regulation of expression of OPN has been observed. OPN is also reported to up-regulate during the osteogenic differentiation of MSCs. Collagen I is an important component of bone extra-cellular matrix. Col1A2 up-regulation has been observed as an early response to a number of different methods of inducing *in vitro* osteogenic differentiation (Florencio-Silva et al., 2015).

In the present study we exposed rat BM-MSCs and AT-MSCs to DC ES and compared osteogenic differentiation behavior in both cell types.

## Materials and Methods

### Groups

We designed our experiments to compare electrically stimulated (ES) versus not stimulated (Control) cell groups. Each group included both BM- and AT-derived rat MSCs, cultivated in osteogenic differentiation supplemented medium, harvested at 3 different time points (three, seven and 14 days) for three different analyses (Calcium deposition staining, viability test and osteogenic gene expression). All experiments were run in triplicate.

### Cell preparation and culture

Sprague-Dawley (SD) rat MSC from bone marrow (RASMX-01001) and adipose tissue (RASMD-01001) were both obtained from Cyagen (CA, USA). Frozen vials of cells were thawed, cultured, and expanded to reach the desired number, based on the cell provider’s instructions. To achieve the appropriate number, cells were cultured at a density of 2.5 × 10^4^ cell/cm^2^ until 80% confluency and then expanded over five passages. Cells from passage 6 were seeded in 6-well cell culture plates (TPP, Trasadingen, Switzerland) at a density of 10^4^ cell/cm^2^ in cell growth medium consisting of Dulbecco’s Modified Eagle Medium (DMEM) + GlutaMAX + 1 g/L D-Glucose + 10% Fetal Calf Serum (FCS) and 1% Penicillin/Streptomycin (10.000 U/ml) all obtained from (Gibco^®^, Gaithersburg, MD, USA), placed in a humidified incubator at 37 °C, 5% CO_2_ and 3% Oxygen (Haque et al., 2013). The culture medium was changed initially, one day after seeding and then twice weekly. From the second day on, the cell growth medium was supplemented with 10^−7^ M dexamethasone, 10 mM β-glycerophosphate, and 0.05 mM ascorbic acid-2-phosphate, all obtained from Sigma-Aldrich (Heidelberg, Germany) (Jaiswal et al., 1997).

### Electrical stimulation of cells

**Figure 1:**
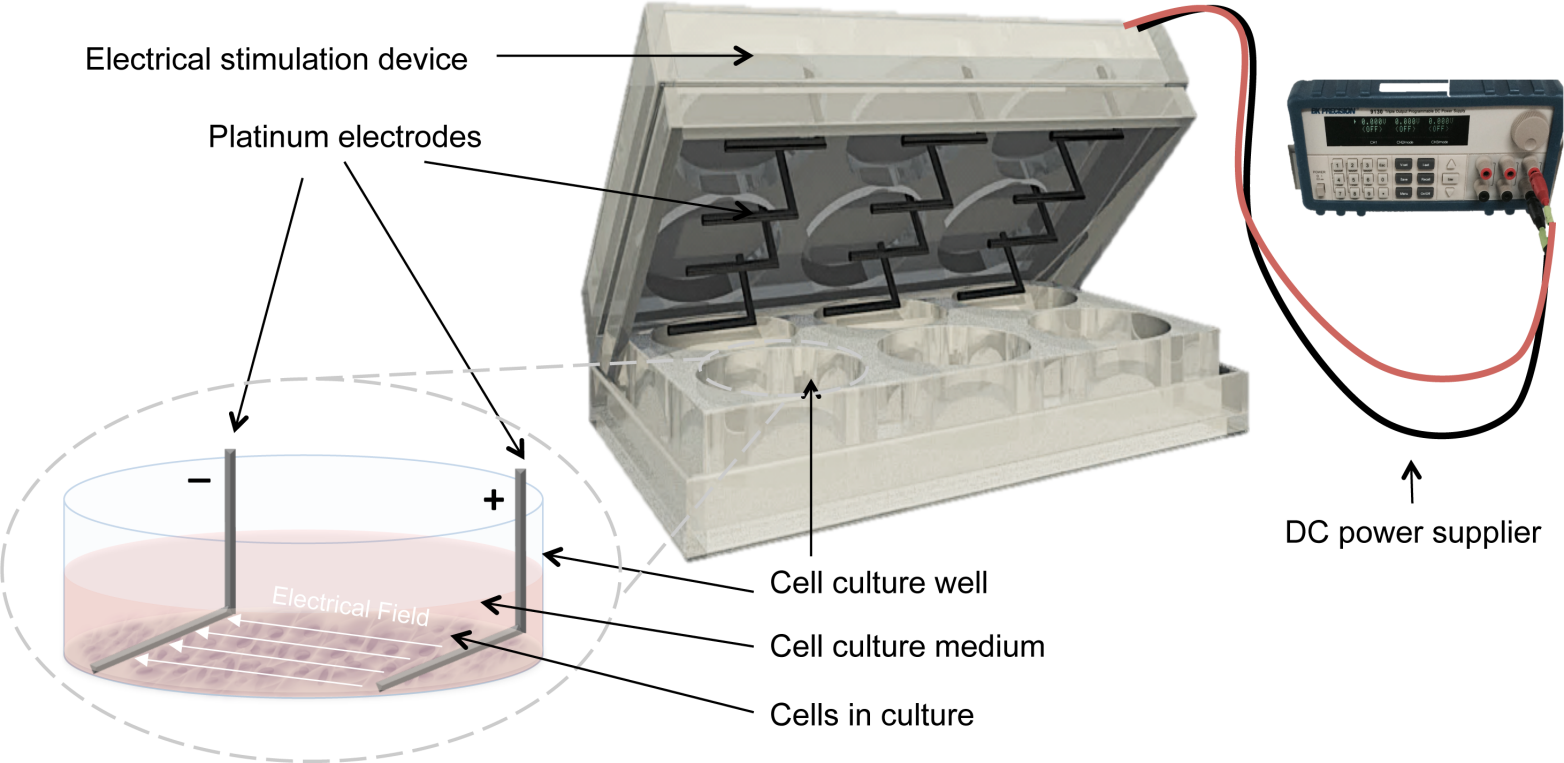
Setup for delivering direct current electrical stimulation to the cells. L-shaped platinum electrodes, 22 mm apart, secured to the lid of a 6-well cell culture plate and connected to a standard DC power supply. The electrodes are in contact with the bottom of the cell culture plate and are fully covered by culture medium.

Electrical stimulation was applied by means of a purpose built DC ES cell culture chamber (Mobini, Leppik & Barker, 2016). Briefly, the chamber consists of L-shaped platinum electrodes, separated by a distance of 22 mm, and secured to the lid of 6-well cell culture plates, (TPP, Trasadingen, Switzerland), and connected to a standard electrical power supply (e.g., Triple Output Programmable DC Power Supplier (Supply-Model 9130; B&K Precision, Yorba Linda, CA, USA)), Fig. 1. For sterilization, electrodes were submerged in 70% ethanol for 10 min and washed by sterile calcium-magnesium free phosphate buffer saline (PBS) (Gibco^®^, Gaithersburg, MD, USA) and finally exposed to UV light overnight. The cells that received electrical stimulation were exposed to 100 mV/mm of DC ES for 1 h per day. All evaluations and assays were performed 24 h after the last exposure.

### Cell viability and activity

To confirm that the oxidation–reduction and electrochemical reactions of the metallic electrodes in the DC ES chamber were not cytotoxic, cell viability and metabolic activity were assessed by 3-(4,5-dimethylthiazol-2-yl)-2,5-diphenyltetrazolium bromide (MTT) assay (Kit I MTT; Roche Diagnostics, Manheim, Germany) after being exposed to ES for 7 and 14 days. Cell culture medium was completely removed and cells were washed twice with 1 mL PBS. One ml of fresh culture medium was added to the wells immediately prior to running the test. Absorbance was read at 550 nm with the reference wavelength of 650 nm, using an Infinite 200PRO NanoQuant device, with TECAN i-control™ software (Tecan, Crailsheim, Germany). We calculated fold change in optical density of electrically stimulated groups, relative to control.

### Osteogenic differentiation

Alizarin Red stains calcium deposits in the cells, indicating the presence of functional osteocytes. Cultured cells were washed twice with PBS and fixed with 4% paraformaldehyde (Sigma Aldrich, München, Germany) solution in PBS for 30 min. Alizarin Red S (Sigma-Aldrich, München, Germany) solution (2% in PBS) was added to the fixed cells, incubated at room temperature for 30 min, and rinsed with deionized water repeatedly. Images were captured with a light microscope (CKX53, cellSens Entry 1.9 Software; Olympus,Tokyo, Japan) at a magnification of 10X.

Temporal osteogenic marker expression was evaluated by two-step Reverse-Transcription quantitative Polymerase Chain Reaction (RT-qPCR) technique. In brief, total RNA was isolated using an Aurum RNA isolation kit (BioRad, München, Germany) according to the manufacturer’s instructions. The quality and quantity of RNA were measured using gel electrophoresis and an Infinite 200PRO NanoQuant device (Tecan, München, Germany). Genomic DNA contamination was removed through digestion using RNase-free DNase-I following the manufacture’s protocol (New England BioLabs GmbH, Germany). DNase-treated RNA samples were reverse transcribed using iScript Select cDNA Synthesis Kit (Bio-Rad, München, Germany) according to the manufacturer’s instructions. The RT-PCR reaction was performed using cDNA equivalent to 1.25 ng RNA and the SsoAdvanced Universal SYBR Green Supermix (BioRad, München, Germany). All samples were amplified in triplicate and PCR was performed using a CFX96 Touch Real Time PCR Detection System (BioRad, München, Germany) under the following conditions: 95 °C for 3 min followed by 40 cycles of 10 s of denaturation at 95 °C and 30 s of annealing and elongation at primer corresponding temperature. The rat gene specific primers used in this study were: Runt-related transcription factor 2 (RunX2, 60 °C, forward CTACTCTGCCGAGCTACGAAAT; reverse TCTGTCTGTGCCTTCTTGGTTC), Osteopontin (SPP1, 62 °C forward GATGAACAGTATCCCGATGCC; reverse TCCAGCTGACTTGACTCATGG) and Collagen type I, alpha 2 protein (Col1A2, 62 °C, forward TTCCCGGTGAATTCGGTCT; reverse ACCTCGGATTCCAA*TAGGACCAG*), all purchased from Sigma Aldrich (Sigma Aldrich, München, Germany). Ribosomal protein P1 (RPLP1, 64 °C forward *GCATCTACTCCGCCCTCATC*, reverse AAGCCAGGCCAGAAAGGTTC) was used as a reference gene (Curtis et al., 2010). A melting curve analysis was applied to ensure the specificity of the PCR; amplification products were also analyzed by gel-electrophoresis.

### Data analysis

All experiments were performed in triplicate and statistical significance of differences between groups was analyzed by one-way ANOVA and student *t*-test using GraphPad Prism (GraphPad Software Inc, La Jolla, CA, USA). Significance level was set at *p* < 0.05. Relative quantification of messenger RNA (mRNA) levels of the target genes was analyzed using the comparative C_T_ (threshold cycle values) method (2^−ΔΔCq^) (Livak & Schmittgen, 2001). The results are presented as relative quantification (RQ), which is expression fold change compared to the calibrator (Our calibrator was the non-stimulated cells, cultivated in growth medium at day 0). Standard deviation (SD) was calculated with the ΔC_q_ value of technical triplicates.

## Results

### Electrical stimulation optimization

**Figure 2:**
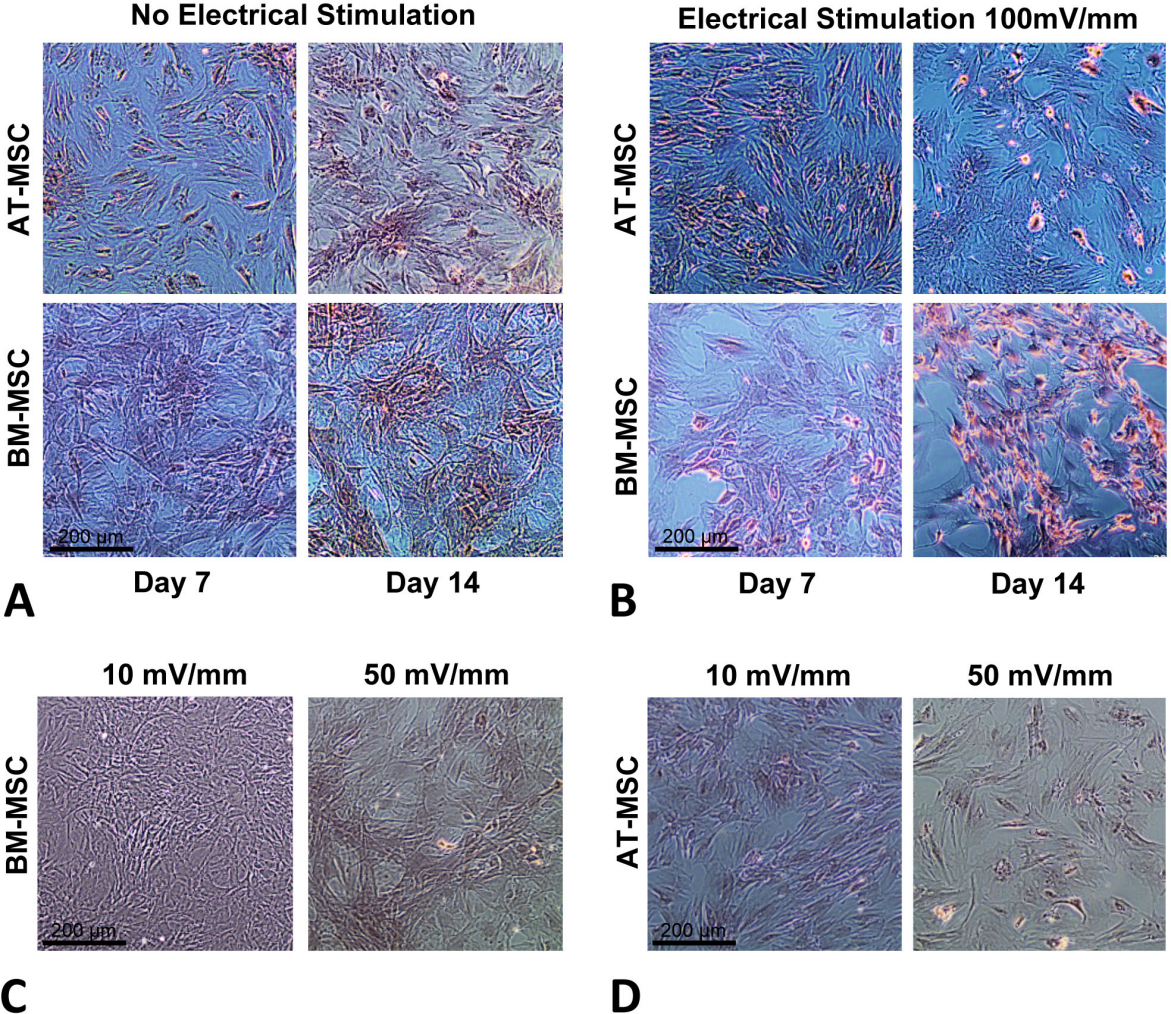
Calcium deposition. Calcium deposition stained using Alizarin Red S for; (A) BM-MSCs and AT-MSCs exposed to no electrical stimulation (controls) at days 7 and 14; (B) BM-MSCs and AT-MSCs exposed to 100 mV/mm of electrical stimulation, at days 7 and 14; Different degrees of staining are visible in electrically stimulated vs. non-stimulated controls; (C) BM-MSCs exposed to 10 and 50 mV/mm of electrical stimulation at day 7; (D) AT-MSCs exposed to 10 and 50 mV/mm of electrical stimulation at day 7 (Magnification = 10×).

In order to optimize the electrical stimulation regime, first we exposed cells in culture to 10, 50, 100, and 200 mV/mm of DC ES for 1 h and found that 200 mV/mm caused cell lysis due to electro-chemical reactions in the vicinity of the electrodes. We found that 10 and 50 mV/mm for 1 h caused no significant changes in proliferation and differentiation (see Figs. 2C and 2D). Based on these preliminary studied we chose 100 mV/mm of DC ES for 1 h per day for the present experiment.

### Cell viability and activity

**Figure 3:**
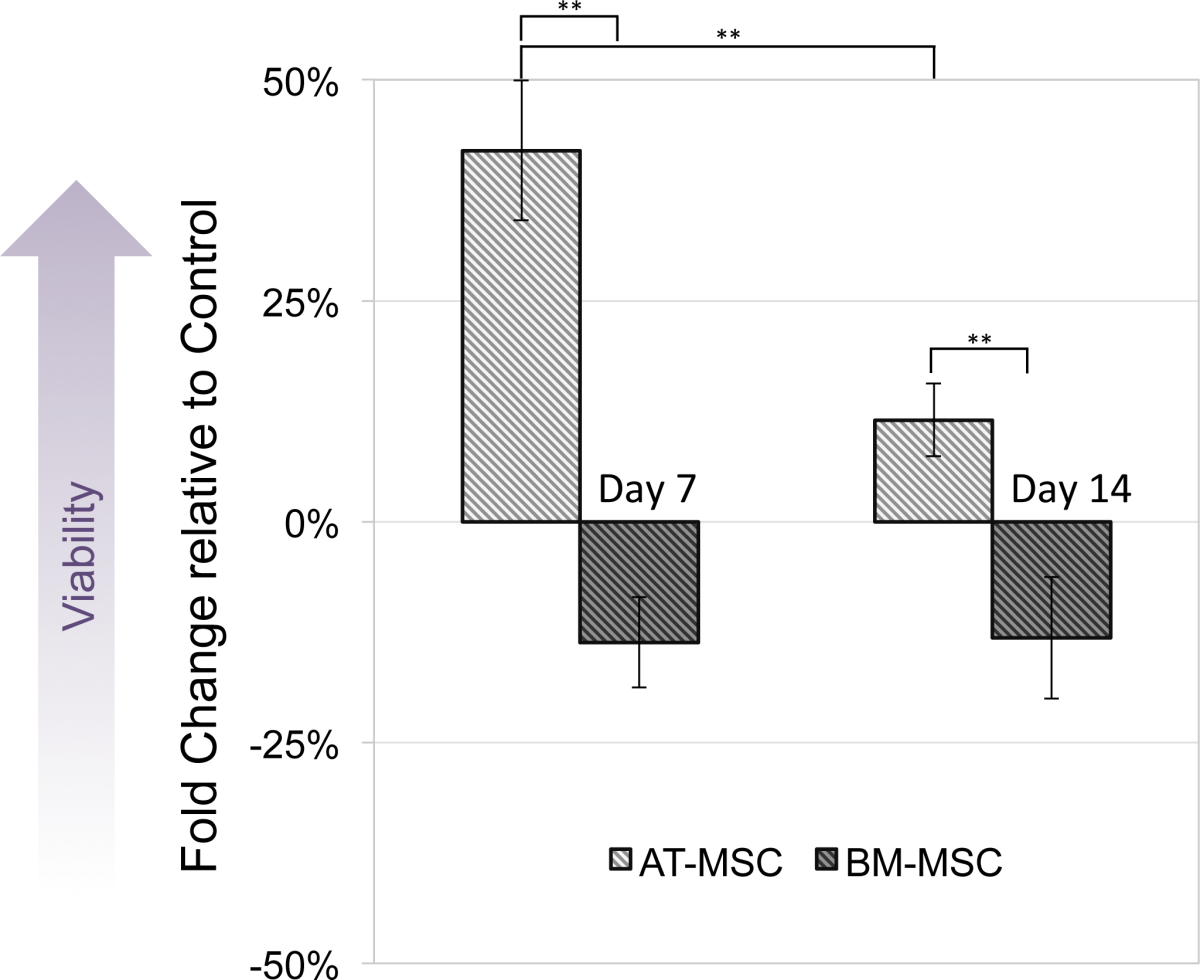
Cell viability. Measured by MTT assay, compared between electrically stimulated and non-stimulated controls. No significant difference in cell viability was detected between ES and non-stimulated control AT- and BM-derived MSC at 7 and 14 days (Values are shown as means ± standard deviations (*n* = 3) ** *p* < 0.01).

MTT assay was performed to compare viability and activity of electrically stimulated cells vs. non-stimulated controls. None of the cells exposed to 10, 50 and 100 mV/mm showed signs of toxicity. Figure 3 shows changes in optical density of groups exposed to 100 mV/mm DC ES compared to controls, indicating differences in cell viability between the different sources in response to electrical stimulation. At day 7, electrically stimulated AT-MSCs had approximately 30 percent increase in cell activity, which can be correlated to higher cell numbers in ES vs. control groups. At this time point BM-MSCs had less cell activity/number in ES vs. control groups. At day 14, the cell number/activity of ES AT-MSCs was slightly higher than controls, while BM-MSCs maintained the same trend.

### Osteogenic differentiation

The influence of electrical stimulation on osteogenic differentiation of rat AT- and BM- MSCs in culture were investigated after seven and 14 days and compared to controls. Figures 2A and 2B shows Alizarin Red S stained calcium deposits in the electrically stimulated (100 mV/mm) cells, initiating at day 7 and becoming significant at day 14, versus controls. Exposure to ES caused distinct changes in morphology and calcium deposition in BM-MCSs at day 14 (Fig. 2B) compared to control cells (Fig. 2A). Calcium deposition in AT-MSCs and BM-MSCs after seven days of exposure to lower (10 and 50 mV/mm) electrical fields, was not significant. However, initial morphological changes in both cell types were present at day 7, in cells exposed to electrical field of 50 mV/mm (Figs. 2C and 2D).

**Figure 4:**
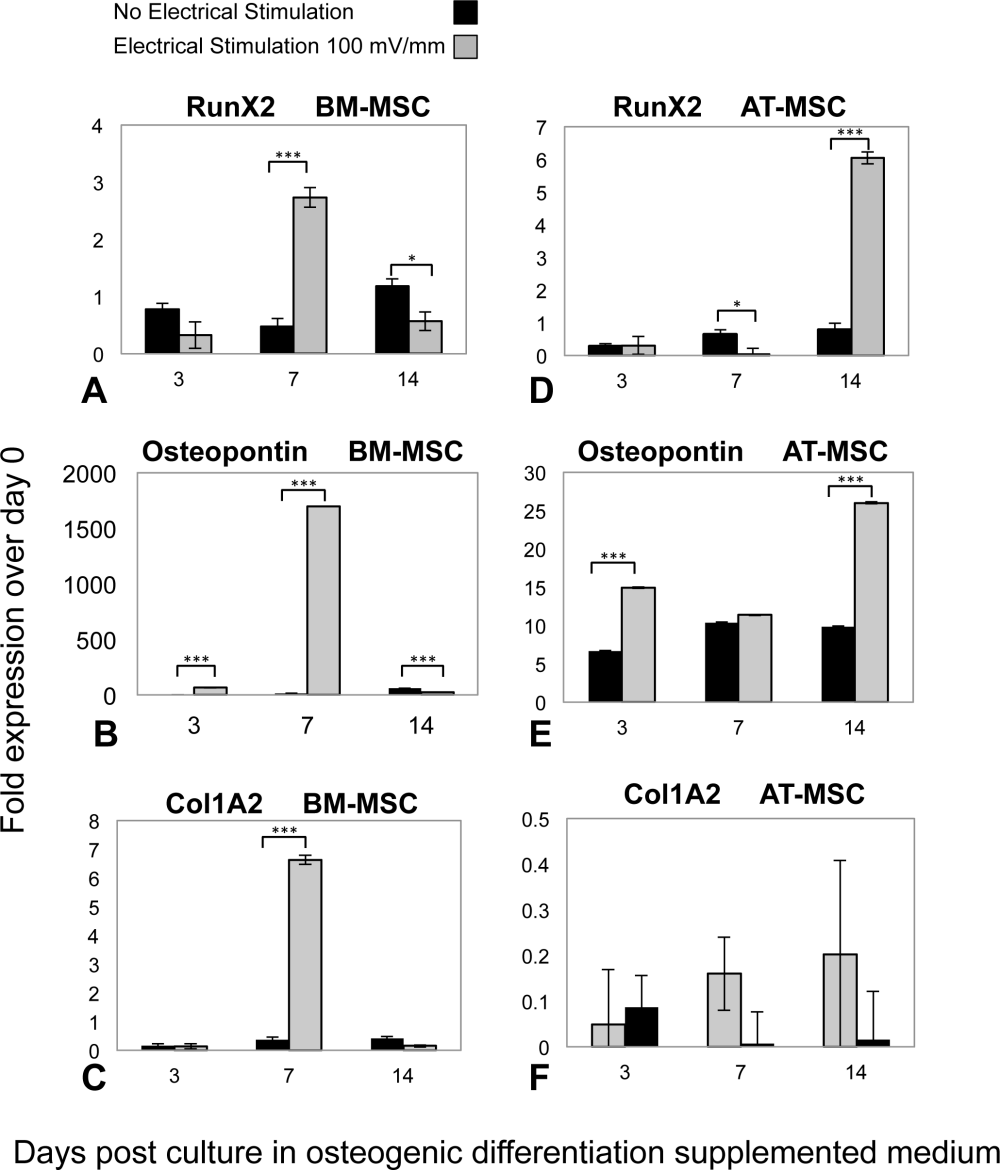
RT-qPCR results. Temporal changes in messenger RNA (mRNA) of (A) Runx2, (B) Osteopontin, (C) Collagen Type1 (Col1A2) in BM-MSCs; (D) Runx2, (E) Osteopontin and (F) Col1A2 in AT-MSCs, in both ES and non-stimulated control cells. Total RNA extracted from cultured cells at days 3, 7 and 14 were transcribed into complementary DNA and subjected to real-time quantitative polymerase chain reaction analysis. The relative mRNA levels are expressed as arbitrary units normalized according to the corresponding levels of Ribosomal Protein P1 mRNA (Values are shown as means ± standard deviations (*n* = 3) * *p* < 0.05; *** *p* < 0.001).

Osteogenic phenotype gene expression was investigated by means of RT-qPCR analysis, in both ES and control groups of AT-MSCs and BM-MSCs at three, seven, and 14 days, (Fig. 4). In both BM- and AT- MSCs, time-dependent gene expression patterns significantly differed in ES vs. control cells. Moreover, there was a difference in expression of osteogenic markers (Runx2, Osteopontin and Col1A2) between the ES BM- and AT-MSCs groups. However, temporal mRNA expressions of the same markers were similar in both AT- and BM-MSC, non-stimulated controls. In BM-MSCs, ES resulted in an increase in the expression of Runx2, Osteopontin and Col1A2 mRNA, three, 1,500 and 14 times, respectively, in comparison to the controls. In the ES AT-MSC groups, the intensification of mRNA levels, Runx2 (six times) and Ostepontin (25 times) was observed on day 14. However there was no difference in expression of collagen type I mRNA (Col1A2) in AT-MSCs in ES and non-stimulated controls.

## Discussion

Early studies exposing bone cells to DC electrical fields were in 1980s when Ferrier et al. (1986) exposed osteoblast-like cells to a 100 mV/mm electrical stimulation and observed cell migration toward the cathode. In 1997 and 1998, changes in TGF-β, BMP-2, and 4 mRNA levels were demonstrated in osteoblasts using capacitive coupling and pulsed electromagnetic field techniques (Bodamyali et al., 1998; Zhuang et al., 1997). Later, several groups described using both indirect (electromagnetic fields or capacitive coupling) and direct (DC) electrical stimulation to promote MSC osteogenic differentiation (Fu et al., 2014; Ross et al., 2015; Sun et al., 2009). For example, in 2010 McCullen et al. reported experiments in which they exposed human AT-MSCs, in osteogenic supplemented medium, to DC ES (100 mV/mm at 1 Hz for 4 h/day) and after 14 days observed a significant increase in mineralization. In another study, rat BM-MSCs were seeded onto conductive polypyrrole films and exposed to 35 mV/mm of DC ES for 4 h and after 14 days significant osteogenic differentiation was observed (Hu et al., 2014).

With the aim of better understanding these DC ES induced changes in MSCs osteogenic differentiation behavior, we exposed rat BM-MSCs and AT-MSCs to 100 mV/mm 1 h/day DC ES for three, seven and 14 days in the presence of osteogenic differentiation supplements in culture medium. The rationale for choosing this stimulation regime was based on reports in the literature (Zhao et al., 2011) and our own previous experiments. In the present study we observed that in most cases ES caused significant changes in osteogenic marker expression patterns both in BM- and AT-derived MSCs when compared to non-stimulated controls. We also found that when BM- and AT-derived MSCs are exposed to DC ES they express osteogenic markers differently. Finally we observed that ES induced elevation of osteogenic markers, Runx2, Osteopontin and Col1A2 at day 7, only in BM-MSC.

We showed that in the presence of DC ES, BM-MSCs and AT-MSCs behave differently as it relates to osteogenic marker expression. These observations could be related to the known differences in osteogenic differentiation capacity between BM- and AT-derived MSC. Namely, studies that suggest AT-MSCs have less osteogenic potential than BM-MSCs (Ratanavaraporn et al., 2009). Moreover, reports indicate that, while a given set of common genes may be involved in early differentiation of MSC from both sources, a different set of genes could be involved in maturation into fully differentiated cells (Liu et al., 2007).

Our results indicate that in control groups (osteogenic supplemented medium, without electrical stimulation), both in BM- and AT-MSCs, only a slight difference exists in temporal expression patterns of Runx2, Osteopontin and Col1A2. However, in the presence of ES, no common expression pattern was detectable. This suggests that ES might activate different cellular mechanisms in BM- vs. AT- MSCs.

Runx2 is known as a master osteogenic transcription factor. Runx2 activates and regulates osteogenesis as the targeted gene of many signaling pathways, including transforming growth factor-beta 1 (TGF-β1), BMP, Wingless type (Wnt), etc. (James, 2013). Osteopontin is an extracellular matrix protein that is highly negatively charged; however regulation of the osteopontin gene is poorly understood. Our findings showed that in the first week ES caused a significant up-regulation of Runx2, Osteopontin and Col1A2 mRNA expression in BM-MSC, while in AT-MSC, the same was observed only in the expression of Runx2 and Osteopontin, after 14 days. We believe that the future of clinical tissue engineering trusts on the techniques and treatments which are able to save time and deliver economic benefits. Electrical stimulation as an old concept with modern applications in medicine appears to be able to shorten the time frame of cell proliferation and differentiation. This means saving time, while reducing costs of cell-based therapies. Moreover, electrical stimulation has the potential to trigger natural procedure of healing and regeneration (Leppik et al., 2015) which could revolutionize the reconstructive and regenerative medicine by implanting electrical stimulation devices which can deliver controlled electrical stimulation doses.

## Conclusions

We have demonstrated that DC ES promotes Runx2, Osteopontin and Col1A2 expression in BM-MSCs already at 7 days. Our results indicate that DC ES effects osteogenic gene expression, in both BM- and AT-MSCs at specific time points. Moreover, these gene expression patterns differ between BM- and AT-derived MSC. These effects and differences should be taken into consideration when applying these two cell sources in tissue engineering applications. Studying ES-induced changes in cellular behavior could lead to methods to control and optimize cell behavior in tissue engineering treatments.

##  Supplemental Information

10.7717/peerj.2821/supp-1 Data S1 Raw Data of MTT

10.7717/peerj.2821/supp-2 Data S2 Raw Data of PCR

## Additional Information and Declarations

### Data Availability

The following information was supplied regarding data availability: The raw data has been supplied as Supplementary Files.
